# The Effect of a Simulation-based Intervention on Emergency Medicine Resident Management of Early Pregnancy Loss

**DOI:** 10.5811/westjem.18596

**Published:** 2024-03-25

**Authors:** Shawna D. Bellew, Erica Lowing, Leah Holcomb

**Affiliations:** * Prisma Health - Upstate, University of South Carolina School of Medicine Greenville, Department of Emergency Medicine, Greenville, South Carolina; † Prisma Health - Upstate, University of South Carolina School of Medicine Greenville, Department of Obstetrics and Gynecology, Greenville, South Carolina; ‡ Clemson University, Department of Public Health Sciences, Clemson, South Carolina

## Abstract

**Background:**

The evaluation of patients with first-trimester vaginal bleeding and concern for early pregnancy loss (EPL) frequently occurs in the emergency department (ED), accounting for approximately 1.6% of all ED visits.^1^ Unfortunately, these patients consistently report negative experiences with ED care.^2^ ^–^ ^8^ In addition to environmental concerns, such as long wait times, patients often describe negative interactions with staff, including a perceived lack of empathy, the use of insensitive language, and inadequate counseling.^2^ ^,^ ^3^ These patients and their partners often view EPL as a traumatic loss of life and commonly experience prolonged grief reactions, including anxiety and depression.^9^ ^–^ ^11^ Poor satisfaction with care has been associated with worse mental health outcomes.^12^ These complaints represent an important opportunity for improvement in emergency medicine (EM) training.^13^

While no published literature to date describes the performance of EM residents in managing patients presenting with EPL, studies suggest that even obstetrics and gynecology (OB/GYN) residents find these interactions challenging.^14^ ^,^ ^15^ Simulation- and didactic-based training has been shown to be beneficial in improving OB/GYN resident EPL counseling and has been associated with improved patient outcomes.^16^ To our knowledge, this has yet to be replicated in EM residency training.

**Objectives:**

We aimed to develop and evaluate a simulation-based educational intervention to improve EM resident management of patients presenting with EPL.

## CURRICULAR DESIGN

**Figure 1.**
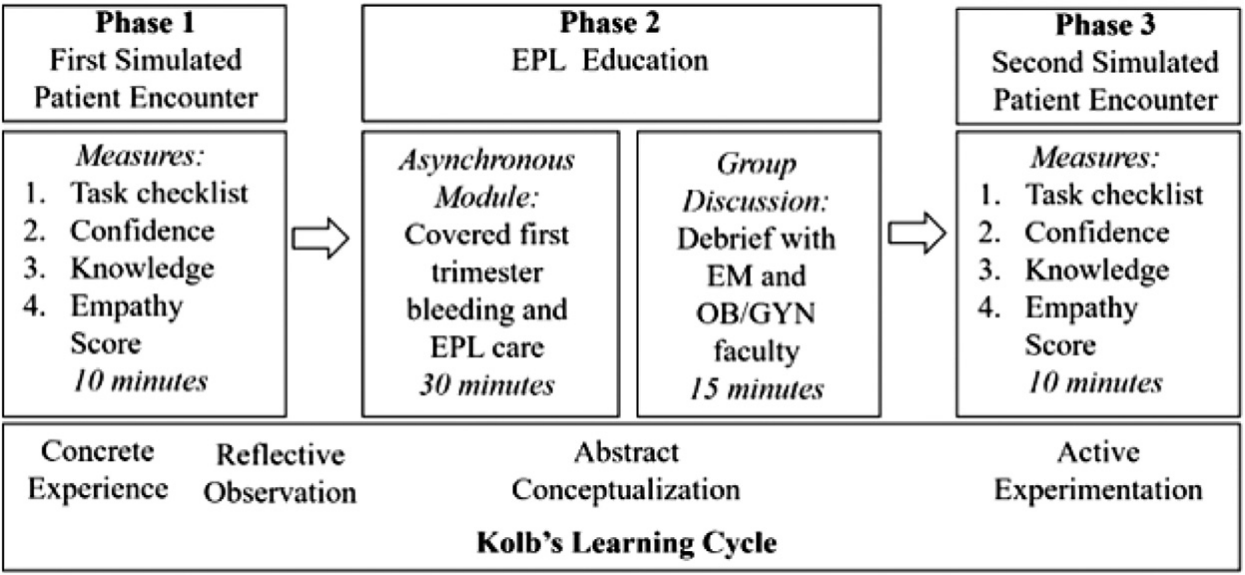
Sequence of an educational intervention for early pregnancy loss counseling. *EPL*, early pregnancy loss.

The educational intervention consisted of three phases (Figure 1) and was designed to optimize learning based on Kolb’s learning cycle.^17^ ^,^ ^18^ Residents were presented with a challenging scenario (concrete experience) and then prompted to reflect on areas for improvement (reflective observation). They then completed an asynchronous module followed by an interactive group discussion (abstract conceptualization). The learning cycle continued through active experimentation via a repeated opportunity to do the simulation, followed by debriefing. This form of repetitive simulation has been shown to be more effective when compared with non-repeated simulation.^19^ ^,^ ^20^

We implemented the intervention in May 2023 and conducted a pre/post study of its immediate impact, which was deemed exempt by our institutional review board. The intervention took place at the simulation center of the affiliated medical school, during the two-hour period typically allotted for monthly resident simulation-based education. Postgraduate year (PGY) 1–3 EM residents were recruited based on a convenience sample including all residents attending simulation that day. The residents were not informed of the topic of the intervention prior to the day of the study, which is typical of our simulation curriculum.

Six standardized patients (SP) were hired to portray patients experiencing EPL. Six volunteer faculty emergency physicians (two men, four women) observed and evaluated the simulations and provided instruction and debriefing. One faculty OB/GYN physician and one faculty emergency physician (both women) co-facilitated the guided group discussion-based education.

First, residents participated in a 10-minute simulated patient encounter in which they were instructed to care for a SP who portrayed a patient who was eight weeks pregnant and presented with vaginal bleeding. Prior to evaluating the patient, each resident was provided with ultrasound results indicating the pregnancy was nonviable (presumably obtained in triage).

Following the encounter, residents individually debriefed with an EM faculty observer. Residents then had 30 minutes to complete an asynchronous online educational module that included content about the assessment of early pregnancy bleeding; diagnosing and managing ectopic pregnancy; preventing alloimmunization; and EPL counseling. Particular attention was paid to optimizing care to address a patient’s physical, emotional, and cognitive needs, a framework recommended by Emond et al.^21^ The module was delivered via an interactive educational platform, Rise 360 Articulate (Articulate, New York, NY).^22^ After completing the module, residents participated in a 15-minute guided group dialog with EM and OB/GYN faculty, discussing best practices and modeling practical communication skills. Facilitators gave examples of how they would address patients in various scenarios to communicate clearly while also using sensitive language.

Following this discussion, residents repeated the same 10-minute simulated patient encounter followed by individual debriefing with EM faculty. The intervention was designed to accommodate up to 24 residents with the resources described.

## IMPACT AND EFFECTIVENESS

To study the immediate impact of the intervention, resident performance was evaluated using four measures: 1) completion of critical actions during the simulation via an 11-item checklist; 2) self-reported confidence; 3) a 10-item multiple-choice test of foundational EPL knowledge; and 4) SP perceptions of resident empathy during the simulation via the modified Jefferson Scale of Empathy (JSE).^23^ ^,^ ^24^ All four evaluative measures were delivered immediately following the initial simulated encounter (Phase 1) and after the final simulation encounter (Phase 3). In addition to these measures, residents were invited to participate in a brief focus group interview, conducted by a non-faculty facilitator (woman), after the intervention to discuss their impressions of the intervention.

Faculty in EM and OB/GYN developed the task checklist to include critical actions and evidence-based best practices in treating patients experiencing EPL. This list was adapted from a checklist employed in a similar study and modified to reflect ED care.^25^ Residents were asked to rate their perceived confidence level from least (1) to most (10) confident regarding the following: knowledge about the evaluation and management of patients with first-trimester bleeding; ability to communicate in a sensitive and empathic manner with patients with EPL; and ability to counsel a patient experiencing EPL regarding what to expect after discharge. They also completed a 10-question multiple-choice test, which EM and OB/GYN faculty developed to assess basic objective knowledge. After each simulated encounter, SPs completed the modified JSE, a validated tool for SP evaluation of clinician empathy and communication. The modified JSE includes five questions on a seven-point Likert scale ranging from strongly disagree (1) to strongly agree (7).^23^ ^,^ ^24^ An outline of the simulated case, the module, and the assessment tools are included in the supplemental material accompanying the online article.

Of the 16 residents who participated, 75% identified as men, and there was relatively equal representation of PGY-1 (31.3%), PGY-2 (37.5%), and PGY-3 (31.3%) residents. Residents improved from pre- to post-intervention across all four evaluative measures (Table 1). Before the intervention, few residents provided information about what to expect after discharge, including the potential pain level, the likelihood of passing tissue, return precautions, and long-term emotional ramifications. After the intervention, residents were significantly more likely to use sensitive language and to include information about expected outcomes and return precautions (Table 1b).

**Table 1.** Resident assessment outcomes pre- to post-intervention.

		Pre	Post	Signed rank	
Measure	Maximum score	Mean (SD)	Mean (SD)	*S*	*P*-value
Performance checklist	11	4.94 (1.80)	9.50 (1.51)	67.0	<.001
Self-confidence	30	20.06 (3.38)	24.69 (3.50)	68.0	<.001
Knowledge	10	5.84 (1.29)	8.00 (1.41)	45.5	<.001
Empathy	35	21.25 (6.04)	28.06 (5.47)	65.5	<.001

**Table 1b.** Resident checklist performance.

	Pre	Post	
Checklist item	n (%)	n (%)	*P*-value
1. Delivers bad news using simple language and with avoidance of non-preferred terms (fetus, embryo)	10 (62.5)	16 (100)	0.03
2. Allows silence for the patient to absorb the news	14 (87.5)	14 (87.5)	1.00
3. Acknowledges patient’s emotions	15 (93.8)	15 (93.8)	1.00
4. Dispels guilt	15 (93.8)	16 (100)	1.00
5. Counsels patient about the amount of expected bleeding	2 (12.5)	11 (68.8)	0.004
6. Counsels patient on expected pain	1 (6.3)	10 (62.5)	0.004
7. Counsels patient on the possibility of passing tissue	2 (12.5)	12 (75.0)	0.006
8. Counsels patient on return for severe bleeding	3 (18.8)	14 (87.5)	0.003
9. Counsels patient on return for fever	2 (12.5)	15 (93.8)	0.001
10. Normalizes emotional ramifications of EPL	5 (31.3)	13 (81.3)	0.008
11. Discusses follow-up plan	10 (62.5)	16 (100)	0.030

*EPL*, early pregnancy loss.

These results indicate that focused training resulted in immediate improvements in resident performance, particularly regarding counseling and communication. Given the positive results of similar interventions undertaken in other learner populations, this immediate impact likely indicates improved ability to care for patients in clinical practice. Verhaeghe et al published the impact of a three-hour in-situ simulation training for OB/GYN residents, which resulted in long-term improvements in psychologic outcomes as well as reduced need for return visits.^16^ As compared to these previous interventions, our curriculum enhanced efficiency by employing an online training module, which covered additional foundational knowledge of early pregnancy bleeding care (including ectopic pregnancy and threatened EPL). This efficiency is particularly important in EM given the breadth of required knowledge.

While the eight residents who participated in the focus group interview generally reported positive feedback, two residents did note that they were confused by the order of the simulation such that they had a diagnosis prior to any interaction with the patient. In the future, this may be ameliorated by providing the residents with more context to the case or simply revising the scenario so that the ultrasound report is received after an initial evaluation and request for imaging. Additionally, the time allotted for the asynchronous module was 30 minutes, but most residents completed it in about 20 minutes, indicating the possibility of additional content or expansion of another aspect of the intervention.

## LIMITATIONS AND CONCLUSION

This study describes resident performance in a simulated patient encounter, and we cannot conclude that this reflects actual clinical care. This study only assessed the impact of the training on learning (Kirkpatrick level 2) and did not attempt to evaluate the residents’ ongoing clinical behavior or its effect on patients.^26^ The study was conducted during one session and, therefore, we cannot infer information about retention of learning. Future work should assess the effect of interventions such as this on clinician behavior and resultant patient outcomes. Faculty evaluators were not blinded during the simulated patient encounters, which could have introduced bias into the evaluation provided via the checklist. This concern is somewhat addressed by the binary nature of the checklist, in which either a task was performed or it was not. Of the assessment tools, only the modified JSE has been externally validated. Creating and validating EM-specific measurement tools for EPL care would ensure more robust data going forward.

“Participants disproportionately identified as men (75%), as compared to the national average in emergency residencies of 62%.^27^ Given the small population from which the study sample was derived, we did not ask participants whether they were cis- or transgender to avoid loss of anonymity. Similarly, we did not ask participants about personal experiences with EPL. Future work could explore the relationship of these characteristics and experiences with clinical performance. Despite these limitations, the results of this study indicate a need for EPL-specific education in EM residency and that a brief, simulation-based intervention was effective in producing immediate improvements. Considering the results of similar studies conducted in other populations, an intervention such as this may result in improved clinical care and long-term patient outcomes in this common, but devastating, presentation.
